# ﻿*Impatienskarenensis* (Balsaminaceae), a new tiny flowered species from Myanmar

**DOI:** 10.3897/phytokeys.243.123303

**Published:** 2024-06-21

**Authors:** Chit Soe Paing, Piyakaset Suksathan, Saroj Ruchisansakun

**Affiliations:** 1 Chit Win Sein Farm, Hpa-an, Kayin State, 13011, Myanmar Chit Win Sein Farm, Hpa-an Hpa-an Myanmar; 2 Queen Sirikit Botanic Garden, Mae Rim, Chiang Mai 50180, Thailand Queen Sirikit Botanic Garden Chiang Mai Thailand; 3 Department of Plant Science, Faculty of Science, Mahidol University, Bangkok 10400, Thailand Mahidol University Bangkok Thailand

**Keywords:** Critically endangered, endemic, Kayin State, limestone species, Southeast Asia

## Abstract

*Impatienskarenensis* (I.sect.Semeiocardium) from Kayin State, Myanmar is described and illustrated here. It is most similar to *I.micromeris*, but differs in having lower petals with outer margins strongly undulate in the lower half (vs. lower petals entire), apex of upper petals acute to obtuse (vs. apex rounded), short stout spur, ± as long as the depth of lower sepal, ca. 2.5 mm long (vs. long attenuate spur, twice as long as the depth of lower sepal, ca. 5 mm long). Its conservation status is also assessed as Critically Endangered.

## ﻿Introduction

A total of 69 native *Impatiens* species have been documented in Myanmar (Kress et al. 2003; Tanaka et al. 2015, 2018, 2022; Ruchisansakun et al. 2017, 2018a, 2018b; Akiyama et al. 2018; Ding et al. 2019; Dakaw Phong San and Ruchisansakun 2022; Myo Min Latt et al. 2023). Amongst these, eight of them belong to Impatienssect.Semeiocardium (Zoll.) S.X.Yu & Wei Wang which is characterised by a 4-lobed capsule and predominantly fused lateral united petals (Ruchisansakun et al. 2015, 2018b; Yu et al. 2015). In August 2022, the first author discovered an unknown species during his expedition in the Kayin State of Myanmar. Here, we present a detailed description, along with colour photographs and additional information, to document this finding.

## ﻿Materials and methods

The new *Impatiens* specimens were collected in Kayin State, Myanmar. The living plants were grown and seeds collected for ex-situ conservation at the Chit Win Sein Farm, while the dried specimen was deposited in the Herbaria (RAF, RANG). The description and line drawings were made from living specimens. The distribution map was made by SimpleMappr (Shorthouse 2010). To determine the IUCN conservation status, the extent of occurrence (EOO) as well as area of occupancy (AOO) were calculated using the GeoCAT (Bachman et al. 2011) and then compared to the IUCN guidelines (IUCN Standards and Petitions Committee 2024).

## ﻿Taxonomy

### ﻿ Impatiens karenensis

Taxon classificationPlantaeEricalesBalsaminaceae

Chit Soe Paing & Ruchis. sp. nov.

46A2AEA7-B14B-5171-9510-458D6880AD5D

urn:lsid:ipni.org:names:77343949-1

Figs 1 , 2 , 3

#### Diagnosis.

*Impatienskarenensis* resembles *I.micromeris*, but differs in having lower petals with outer margins strongly undulate in the lower-half (vs. lower petals entire), apex of upper petals acute to obtuse (vs. rounded), short stout spur, ± as long as the depth of lower sepal, ca. 2.5 mm long (vs. long attenuate spur, twice as long as the depth of lower sepal, ca. 5 mm long).

**Figure 1.**
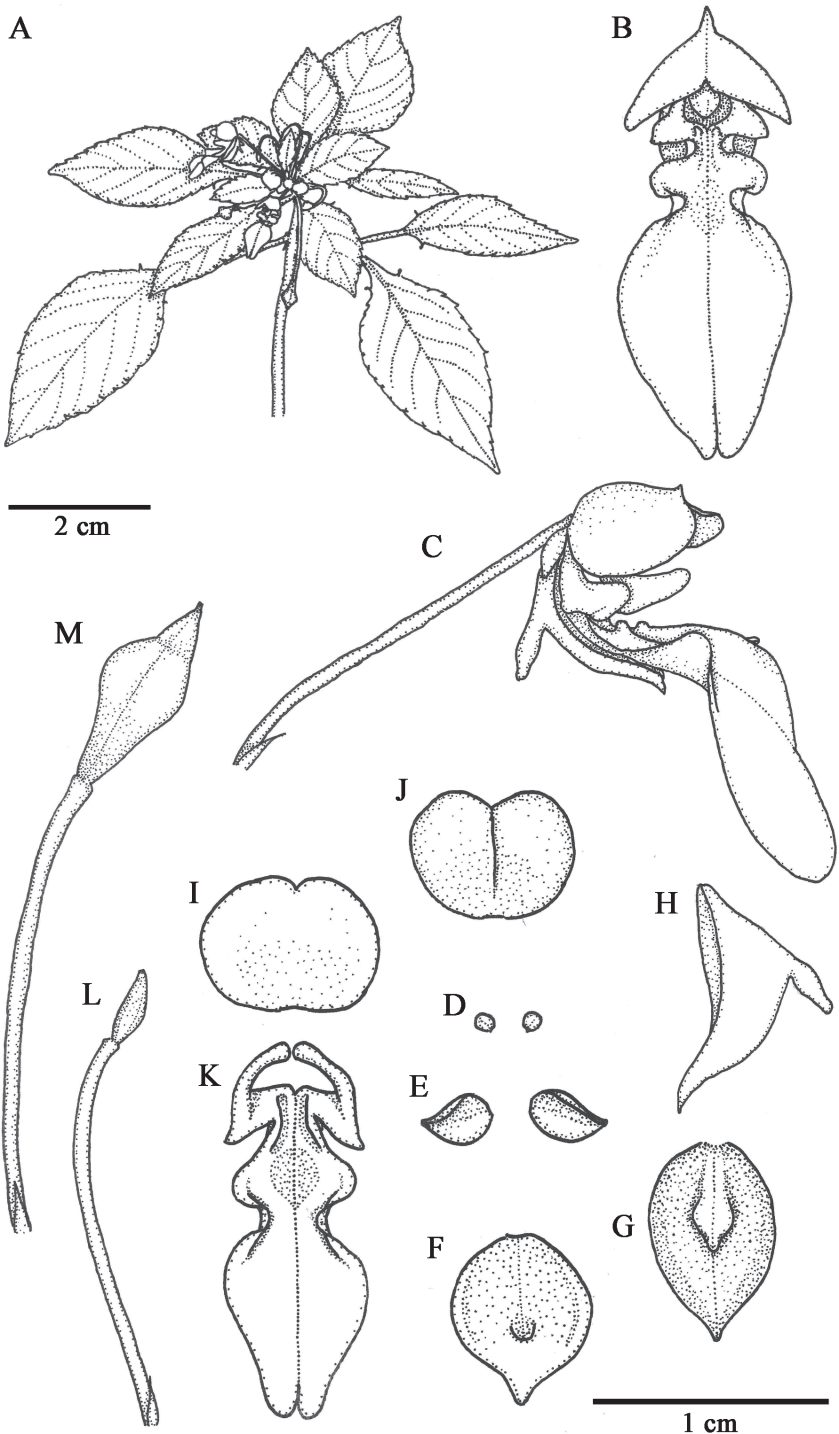
*Impatienskarenensis* Chit Soe Paing & Ruchis **A** habit **B** flower, front view **C** flower, side view **D** inner lateral sepals **E** outer lateral sepals **F–H** lower sepal **I–J** dorsal petal **K** lateral united petals **L** ovary, pedicel and bract **M** fruit (from *Chit Soe Paing 002*). Drawn by S. Ruchisansakun.

#### Type.

Myanmar. Kayin State (Karen State), Hpa-an, Ta Yoke Hla (Kawt Kyaik), 16°50'31.4"N, 97°37'10.4"E, 100–150 m a.s.l., 16 Oct 2023, *Chit Soe Paing 002* (holotype RAF!, isotype RAF, RANG).

**Figure 2.**
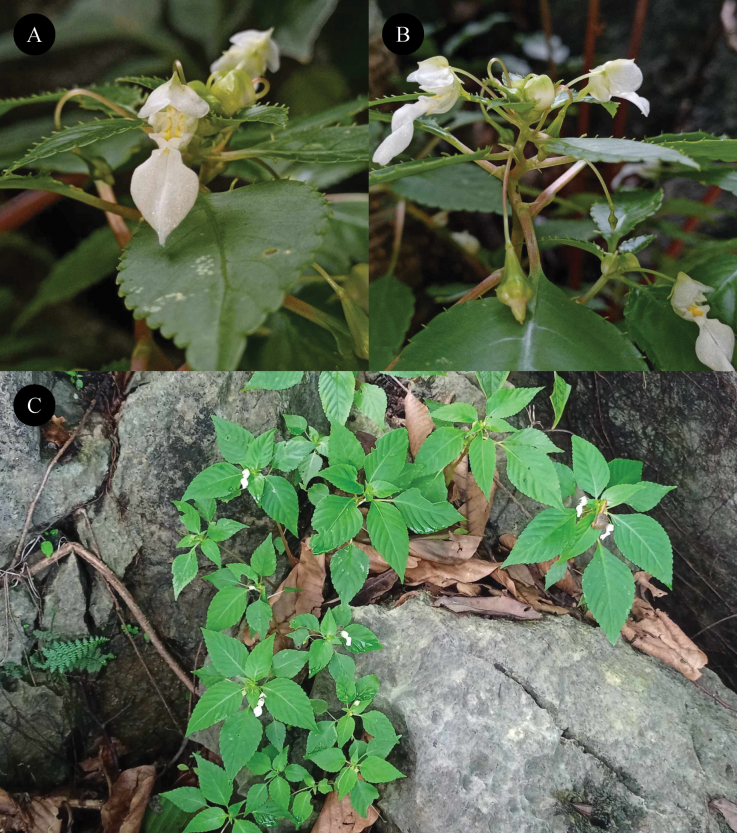
*Impatienskarenensis* Chit Soe Paing & Ruchis. *in vivo***A** flower, front view **B** flowers, side view **C** habit. Photographed by Chit Soe Paing.

#### Description.

Lithophytic annual herbs, 50–70 cm tall. ***Stems*** erect, laxly branched near the top, green to red to purple with red dots, glabrous; hypocotyl elongate, 1–2 cm in diam., epicotyl slightly zigzag in the upper parts. ***Leaves*** spirally arranged, congested on the top when young; petiole ca. 2 cm long, ca. 1.3 mm in diameter, green to reddish-green; lamina ovate, 4–5 × 2.3–2.5 cm, apex acute, base cuneate, green above, paler green below, glabrous, margin serrate with teeth, with a pair of linear glands at the margin near the base, lateral main veins 4–6 pairs. Inflorescence axillary, 2-flowered fascicle; bracts linear-lanceolate, ca. 2 mm long. Flowers white with yellow patch and two yellow streaks at the lip base, 19–19.5 × 6–7 mm, 7–8 mm deep; pedicel 1.6–1.7 cm long, green to reddish-green, glabrous. Lateral sepals 4; inner pair ovate, 0.6–0.7 × 0.5–0.6 mm, apex round, green, glabrous; outer pair free, ovate, ca. 3 × 2 mm, apex acute, green, glabrous. Lower sepal navicular, 7–9 × 5–5.5 mm, 2–3 mm deep, light green, apex acute and mucronate, abruptly constricted into a short strait green spur, ca. 2.5 mm long. Dorsal petal 5.4–5.6 × 6.5–7.5 mm, broadly ovate to broadly obovate to suborbicular, green at the margin and apex, apex emarginate-mucronate, mid-vein crested, ca. 0.5 mm high. Lateral united petals connate, white with yellow mark in the middle at ca. ¼ from the base, clawed to 2–3 mm long; upper petals, 3–3.5 × 1.5–2 mm, ovate to triangular, apex acute to obtuse; lower petals 1.4–1.52 × 0.34–0.35 cm, narrowly ovate in outline, apex obtuse-slightly bilobed, base with two small triangular projections, outer margins strongly undulate in the lower half. Stamens ca. 3 mm long. Ovary 4-loculate, ca. 3 mm long, green, glabrous. ***Fruits*** clavate, 4-lobed, ca. 9 mm long, green, glabrous with green to red pedicel. Seeds unknown.

#### Habitat and phenology.

Grows in open areas on small limestone mountains, 100–400 m a.s.l. Flowering. August–October, fruiting October–November.

#### Distribution.

**Figure 3.**
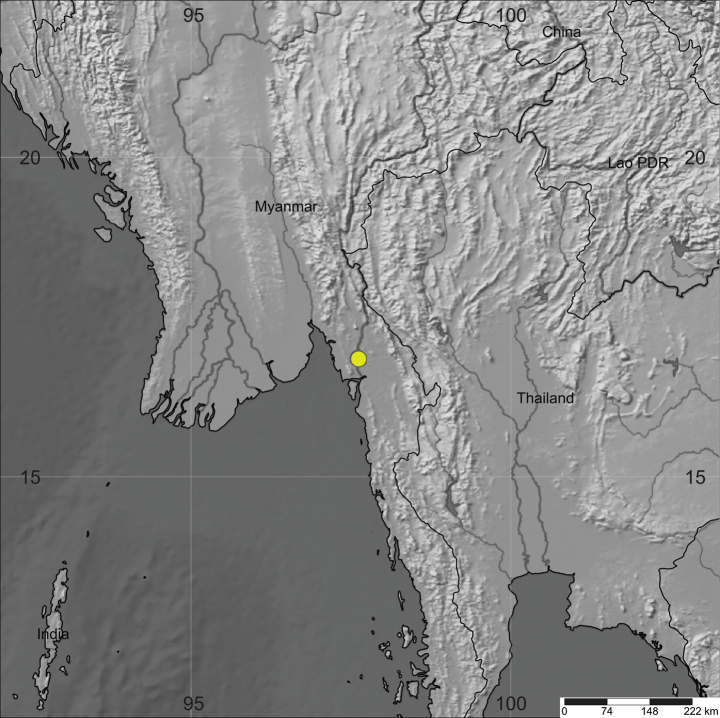
The distribution of *Impatienskarenensis* Chit Soe Paing & Ruchis. (SimpleMappr, Shorthouse 2010).

Endemic to Myanmar. This species is only known from the two localities, around 2 km apart (Fig. 3).

#### Etymology.

The specific epithet is derived from the former name of the state of its type locality “Karen”.

#### Conservation status proposed.

*Impatienskarenensis* is currently found at two locations outside of the protected area. This species has a limited extent of occurrence (EOO) and area of occupancy (AOO), spanning less than 10 km^2^ (GeoCAT, Bachman et al. (2011)). The population of mature individuals is known to fluctuate. Moreover, the habitat quality is consistently declining because of warmer and drier conditions, particularly by El Niño, coupled with invasive species encroachment. Based on these significant threats, we strongly recommend designating *Impatienskarenensis* as Critically Endangered (CR; B1+B2ac(iv)) according to the IUCN Categories and Criteria (IUCN Standards and Petitions Committee 2024).

#### Note.

*Impatienskarenensis* shares similarities with *I.micromeris* and other small *Semeiocardium* species. It also bears resemblance to *I.suksathanii*, but differs in having undulate margin lower petals (vs. entire margin lower sepals), white flower (vs. pink or yellow flower), ovate leaves (vs. linear to narrowly elliptic leaves) (Suksathan and Ruchisansakun 2022).

### ﻿Key to species of Impatienssect.Semeiocardium in Myanmar

**Table:** 

1	Flower very small, less than 2 cm long	**2**
–	Flower much larger, more than 3 cm long	**4**
2	Flowers non-resupinate, very small, up to 10 mm, spur facing upwards and incurved	***I.capillipes***
–	Flowers resupinate, larger than 10 mm, spur downwards, incurved or straight	**3**
3	Lower petals with outer margins strongly undulate in the lower half; upper petals apex acute	***I.karenensis***
–	Lower petals with entire outer margins; upper petals apex rounded	***I.micromeris***
4	Lateral united petals free; upper pair of lateral sepals linear	***I.laevigata***
–	Lateral united petals connate; upper pair of lateral sepals ovate to elliptic or absent	**5**
5	Perennial shrub, (45–)150–300 cm tall; basal part of stems grey, 10–80 mm in basal diam	**6**
–	Annual herb, 15–40(–100) cm tall; stem light green to red to purple, 2–7(–22) mm diam	**7**
6	Pedicels shorter than petioles of the subtending leaves; lower petals without orange to red longitudinal lines	***I.parishii***
–	Pedicels longer than petioles of the subtending leaves; lower petals with orange to red longitudinal lines	***I.kerriae***
7	Lower sepal navicular	***I.lobbiana***
–	Lower sepal deeply bucciniform	**8**
8	Flowers zygomorphic; pedicel pendulous; spur hooked, shorter than 6 mm	***I.psittacina***
–	Flowers asymmetric; pedicel erect; spur curved, longer than 10 mm	***I.tanintharyiensis***

## Supplementary Material

XML Treatment for Impatiens karenensis

## ﻿Additional information

### Author ORCIDs

Piyakaset Suksathan https://orcid.org/0000-0003-4772-4914

Saroj Ruchisansakun https://orcid.org/0000-0002-7022-8831

### Data availability

All of the data that support the findings of this study are available in the main text.
